# Supplementary material for: Finding the Common Single-Nucleotide Polymorphisms in Three Autoimmune Diseases and Exploring Their Bio-Function by Using a Reporter Assay
Source: Biomedicines. 2023 Aug 30;11(9):2426. doi: 10.3390/biomedicines11092426 (PMC10526089; doi:10.3390/biomedicines11092426)
Supplement: Supplementary file 1 [file biomedicines-11-02426-s001.zip › biomedicines-2530713-supplementary.pdf]

Supplement Table S1. The complete data of genotype analysis in GO cases and healthy controls.

SNP	Genotype	Genotype frequency		Odds ratio 95 % CI.	P value	Q value
		Patient (n)	Control (n)			
**CTLA4**						
rs11571315	CC vs. CT vs. TT				0.006*	0.0720
	TT	28	15	Ref.	1.000	
	CT	11	18	0.327 (0.123-0.870)	0.023*	0.1712
	CC	1	7	0.077 (0.009-0.682)	0.015*	0.1675
	TT vs. CT + CC			0.257 (0.101-0.652)	0.004*	0.0766
	TT + CT vs. CC			0.121 (0.014-1.034)	0.057	0.2634
rs733618	CC vs. CT vs. TT				0.011*	0.0977
	CC	13	5	Ref.	1.000	
	CT	21	18	0.449 (0.134-1.502)	0.189	0.4715
	TT	6	17	0.136 (0.034-0.545)	0.003*	0.0766
	CC vs. CT + TT			0.297 (0.094-0.934)	0.032*	0.1787
	CC + CT vs. TT			0.239 (0.082-0.696)	0.007*	0.1173
rs4553808	AA vs. AG vs. GG				0.019*	0.0977
	AA	37	29	Ref.	1.000	
	AG	3	11	0.214 (0.055-0.838)	0.019*	0.1675
	GG	0	0	NA	NA	
	AA vs. AG+GG			0.214 (0.055-0.838)	0.019*	0.1675
	AA+AG vs. GG			NA	NA	
rs11571316	GG vs. AG vs. AA				0.056	0.1833
	GG	32	22	Ref.	1.000	
	AG	7	15	0.321 (0.112-0.916)	0.030*	0.1787
	AA	1	3	0.229 (0.022-2.349)	0.305	0.6089
	GG vs. AG+AA			0.306 (0.113-0.826)	0.017*	0.1675
	GG+AG vs. AA			0.316 (0.031-3.178)	0.615	0.8171
rs62182595	GG vs. AG vs. AA				0.094	0.2417
	GG	37	30	Ref.	1.000	
	AG	3	9	0.270 (0.065-1.088)	0.066	0.2786
	AA	0	1	NA	0.456	0.7624
	GG vs. AG+AA			0.243 (0.061-0.964)	0.066	0.2786
	GG+AG vs. AA			NA	1.000	1.0000
rs16840252	CC vs. CT vs. TT				0.019*	0.0977

	CC	37	29	Ref.	1.000	
	CT	3	11	0.214 (0.055-0.838)	0.019*	0.1675
	TT	0	0	NA	NA	
	CC vs. CT+TT			0.214 (0.055-0.838)	0.019*	0.1675
	CC+CT vs. TT			NA	NA	
rs5742909	CC vs. CT vs. TT				0.077	0.2132
	CC	36	30	Ref.	1.000	
	CT	4	10	0.333 (0.095-1.171)	0.077	0.2786
	TT	0	0	NA	NA	
	CC vs. CT + TT			0.333 (0.095-1.171)	0.077	0.2786
	CC + CT vs. TT			NA	NA	
rs231775	GG vs. AG vs. AA				0.114	0.2565
	GG	24	16	Ref.	1.000	
	AG	15	17	0.588 (0.230-1.505)	0.267	0.5857
	AA	1	5	0.133 (0.014-1.250)	0.079	0.2786
	GG vs. AG+AA			0.485 (0.197-1.196)	0.114	0.3321
	GG+AG vs. AA			0.169 (0.019-1.522)	0.104	0.3097
rs3087243	GG vs. AG vs. AA				0.311	0.4868
	GG	28	22	Ref.	1.000	
	AG	11	15	0.567 (0.221-1.501)	0.257	0.5740
	AA	1	3	0.262 (0.025-2.695)	0.326	0.6331
	GG vs. AG+AA			0.524 (0.209-1.314)	0.166	0.4362
	GG+AG vs. AA			0.316 (0.031-3.178)	0.615	0.8171
rs11571319	GG vs. AG vs. AA				<0.001*	0.0178
	GG	34	16	Ref.	1.000	
	AG	6	23	0.123 (0.042-0.360)	<0.001*	0.0302
	AA	0	1	NA	0.333	0.6375
	GG vs. AG+AA			0.118 (0.040-0.344)	<0.001*	0.0302
	GG+AG vs. AA			NA	1.000	1.0000
**CD28**						
rs1879877	GG vs. GT vs. TT				0.181	0.3366
	TT	21	13	Ref.	1.000	
	GT	11	17	0.401 (0.144-1.118)	0.078	0.2786

	GG	7	9	0.481 (0.144-1.608)	0.231	0.5431
	TT vs. GT + GG			0.429 (0.171-1.071)	0.068	0.2786
	TT + GT vs. GG			0.729 (0.241-2.204)	0.575	0.8171
rs3181096	CC vs. CT vs. TT				0.125	0.2647
	CC	27	24	Ref.	1.000	
	CT	12	9	1.185 (0.426-3.301)	0.745	0.9105
	TT	1	6	0.148 (0.017-1.320)	0.104	0.3097
	CC vs. CT+TT			0.770 (0.306-1.941)	0.580	0.8171
	CC+CT vs. TT			0.141 (0.016-1.232)	0.057	0.2634
rs3181097	GG vs. AG vs. AA				<0.001*	0.0178
	AA	15	6	Ref.	1.000	
	AG	24	18	0.533 (0.173-1.646)	0.271	0.5857
	GG	1	15	0.027 (0.003-0.249)	<0.001*	0.0302
	GG vs. AG+ AA			0.303 (0.103-0.892)	0.026*	0.1742
	GG +AG vs. AA			0.041 (0.005-0.331)	<0.001*	0.0302
rs3181098	GG vs. AG vs. AA				0.055	0.1833
	GG	27	25	Ref.	1.000	
	AG	13	9	1.337 (0.488-3.669)	0.572	0.8171
	AA	0	5	NA	0.053	0.2630
	GG vs. AG+AA			0.860 (0.339-2.180)	0.750	0.9105
	GG+AG vs. AA			NA	0.026*	0.1742
**TNFSF4**						
rs1234314	GG vs. CG vs. CC				0.364	0.5242
	GG	12	16	Ref.	1.000	
	CG	26	20	1.733 (0.671-4.476)	0.254	0.5740
	CC	2	4	0.667 (0.104-4.261)	1.000	1.0000
	GG vs.CG+ CC			1.556 (0.616-3.927)	0.348	0.6477
	GG +CG vs. CC			0.474 (0.082-2.746)	0.674	0.8614
rs45454293	CC vs. CT vs. TT				0.187	0.3366
	CC	27	30	Ref.	1.000	
	CT	13	8	1.806 (0.649-5.021)	0.255	0.5740
	TT	0	2	NA	0.495	0.7624
	CC vs. CT+TT			1.444 (0.545-3.828)	0.459	0.7624
	CC +CT vs. TT			NA	0.494	0.7624
**PD1**						
rs5839828	66 vs. 67 vs. 77				0.400	0.5538
	66	14	18	Ref.	1.000	

	67	15	15	1.286 (0.473-3.495)	0.622	0.8171
	77	8	4	2.571 (0.641-10.310)	0.176	0.4535
	66 vs. 67 + 77			1.556 (0.617-3.928)	0.348	0.6477
	66 + 67 vs. 77			2.276 (0.620-8.349)	0.207	0.5043
rs36084323	TT vs. CT vs. CC				0.110	0.2565
	TT	8	15	Ref.	1.000	
	CT	18	17	1.985 (0.671-5.871)	0.212	0.5073
	CC	11	5	4.125 (1.057-16.097)	0.037*	0.1983
	TT +CT vs. CC			2.472 (0.890-6.864)	0.079	0.2786
	TT vs. CT+ CC			2.708 (0.835-8.785)	0.090	0.2974
rs10204525	TT vs. CT vs. CC				0.075	0.2132
	TT	17	27	Ref.	1.000	
	CT	16	11	2.310 (0.868-6.146)	0.091	0.2974
	CC	6	2	4.765 (0.860-26.383)	0.118	0.3364
	TT +CT vs. CC			2.688 (1.076-6.715)	0.032*	0.1787
	TT vs. CT+ CC			3.455 (0.652-18.294)	0.154	0.4211
rs41386349	GG vs. AG vs. AA				0.554	0.6434
	GG	27	27	Ref.	1.000	
	AG	12	10	1.200 (0.444-3.244)	0.719	0.9089
	AA	1	3	0.333 (0.033-3.410)	0.612	0.8171
	GG vs. AG+ AA			1.000 (0.392-2.549)	0.409	0.7325
	GG +AG vs. AA			0.316 (0.031-3.178)	0.615	0.8171
rs6705653	CC vs. CT vs. TT				0.521	0.6252
	CC	22	25	Ref.	1.000	
	CT	16	11	1.653 (0.634-4.308)	0.302	0.6089
	TT	2	3	0.758 (0.116-4.958)	1.000	1.0000
	CC vs. CT+ TT			1.461 (0.592-3.606)	0.410	0.7325
	CC +CT vs. TT			0.632 (0.100-4.002)	0.675	0.8614
rs2227982	AA vs.AG vs.GG				0.346	0.5190
	AA	10	14	Ref.	1.000	
	AG	19	19	1.400 (0.499-3.925)	0.522	0.7949
	GG	11	6	2.567 (0.711-9.266)	0.146	0.4076
	AA vs. AG+GG			1.680 (0.637-4.430)	0.292	0.6089

	AA+AG vs. GG			2.086 (0.686-6.348)	0.190	0.4715
rs2227981	GG vs. AG vs. AA				0.220	0.3771
	GG	21	25	Ref.	1.000	
	AG	18	11	1.948 (0.755-5.028)	0.166	0.4362
	AA	1	3	0.397 (0.038-4.105)	0.621	0.8171
	GG vs. AG+ AA			1.616 (0.656-3.981)	0.296	0.6089
	GG +AG vs. AA			0.308 (0.031-3.094)	0.359	0.6590
**ICOS**						
rs11571305	GG vs. AG vs. AA				0.512	0.6252
	GG	12	15	Ref.	1.000	
	AG	26	25	1.300 (0.509-3.317)	0.583	0.8171
	AA	1	0	NA	0.464	0.7624
	GG vs. AG+AA			1.350 (0.531-3.435)	0.528	0.7950
	GG+AG vs. AA			NA	0.494	0.7624
rs11889352	AA vs. AT vs. TT				0.045*	0.1800
	AA	15	23	Ref.	1.000	
	AT	17	16	1.629 (0.635-4.183)	0.309	0.6089
	TT	7	1	10.733 (1.197-96.283)	0.020*	0.1675
	AA vs. AT+TT			2.165 (0.881-5.322)	0.090	0.2974
	AA+AT vs. TT			8.531 (0.997-73.006)	0.029*	0.1787
rs11883722	GG vs. AG vs. AA				0.237	0.3878
	GG	12	13	Ref.	1.000	
	AG	19	24	0.858 (0.319-2.305)	0.761	0.9105
	AA	8	3	2.889 (0.618-13.496)	0.277	0.5892
	GG vs. AG+AA			1.083 (0.419-2.798)	0.869	1.0000
	GG+AG vs. AA			3.183 (0.777-13.038)	0.095	0.3031
rs10932029	TT vs. CT vs. CC				0.868	0.9469
	TT	31	31	Ref.	1.000	
	CT	7	5	1.400 (0.401-4.891)	0.597	0.8171
	CC	2	2	1.000 (0.132-7.555)	1.000	1.0000
	TT vs. CT + CC			1.286 (0.425-3.886)	0.656	0.8534
	TT + CT vs. CC			0.947 (0.127-7.087)	1.000	1.0000
rs10183087	AA vs. AC vs. CC				0.163	0.3260

	AA	21	10	Ref.	1.000	
	AC	14	1	6.667 (0.766-58.040)	0.074	0.2786
	CC	3	1	1.429 (0.132-15.516)	1.000	1.0000
	AA vs. AC+CC			4.048 (0.779-21.020)	0.100	0.3097
	AA+AC vs. CC			0.943 (0.089-10.010)	1.000	1.0000
rs10932035	GG vs. AG vs. AA				0.015*	0.0977
	GG	18	3	Ref.	1.000	
	AG	15	17	0.147 (0.036-0.600)	0.004*	0.0766
	AA	5	2	0.417 (0.054-3.221)	0.574	0.8171
	GG vs. AG+AA			0.175 (0.044-0.693)	0.008*	0.1191
	GG+AG vs. AA			1.515 (0.268-8.558)	1.000	1.0000
rs10932036	AA vs. AT vs. TT				0.481	0.6210
	AA	33	34	Ref.	1.000	
	AT	6	3	2.061 (0.476-8.929)	0.481	0.7624
	TT	0	0	NA	0.493	0.7624
	AA vs. AT+TT			2.061 (0.476-8.929)	0.481	0.7624
	AA+AT vs. TT			NA	0.487	0.7624
rs4404254	TT vs. CT vs. CC				0.995	1.000
	TT	22	21	Ref.	1.000	
	CT	14	14	0.955 (0.368-2.473)	0.924	1.0000
	CC	3	3	0.955 (0.173-5.269)	1.000	1.0000
	TT vs. CT + CC			0.955 (0.388-2.347)	0.919	1.0000
	TT + CT vs. CC			0.972 (0.184-5.147)	1.000	1.0000
rs10932037	CC vs. CT vs. TT				0.737	0.8291
	CC	33	34	Ref.	1.000	
	CT	6	4	1.545 (0.400-5.978)	0.737	0.9105
	TT	0	0	NA	NA	
	CC vs. CT+ TT			1.545 (0.400-5.978)	0.737	0.9105
	CC +CT vs. TT			NA	NA	
rs10932038	AA vs.AG vs.GG				0.483	0.6210
	AA	33	33	Ref.	1.000	

	AG	6	3	2.000 (0.461-8.677)	0.483	0.7624
	GG	0	0	NA	NA	
	AA vs. AG+GG			2.000 (0.461-8.677)	0.483	0.7624
	AA+AG vs. GG			NA	NA	
rs1559931	GG vs. AG vs. AA				0.922	0.9762
	GG	22	21	Ref.	1.000	
	AG	14	12	1.114 (0.420-2.955)	0.829	0.9831
	AA	3	2	1.432 (0.217-9.444)	1.000	1.0000
	GG vs. AG+AA			1.157 (0.459-2.927)	0.755	0.9105
	GG+AG vs. AA			1.375 (0.216-8.749)	1.000	1.0000
rs4675379	GG vs. CG vs. CC				0.042*	0.1800
	GG	27	9	Ref.	1.000	
	CG	10	12	0.278 (0.090-0.859)	0.023*	0.1712
	CC	2	0	NA	1.000	1.0000
	GG vs. CG+CC			0.333 (0.111-1.001)	0.047*	0.2422
	GG+CG vs. CC			NA	0.537	0.7995

NA: not applicable; Ref: reference; CI: confidence interval. *:$p<0.05$. rs10932035 of ICOS and rs3181097 of CD28 were deviated from HWE.
